# Detecting the Second Mesiobuccal Canal in Maxillary Molars in a Saudi Arabian Population: A Micro-CT Study

**DOI:** 10.1155/2019/9568307

**Published:** 2019-03-13

**Authors:** Khalid Alfouzan, Abdulmohsen Alfadley, Lubna Alkadi, Abdullah Alhezam, Ahmed Jamleh

**Affiliations:** ^1^Restorative and Prosthetic Dental Sciences, College of Dentistry, King Saud bin Abdulaziz University for Health Sciences, National Guard Health Affairs, Riyadh, Saudi Arabia; ^2^King Abdullah International Medical Research Centre, National Guard Health Affairs, Riyadh, Saudi Arabia

## Abstract

The aim of this study was to determine MB2 canal detectability in maxillary first and second molars obtained from a Saudi population using micro-CT. Maxillary first (*n* = 35) and second (*n* = 30) molars were scanned with micro-CT technology. The number of canals was recorded, and in case of having more than one canal, the level of extracanal detection was analyzed. The presence of extracanal was categorized based on the level they were first detected. Among the maxillary first molars, 28 (80%) and six (17%) teeth had two and three MB canals, respectively. Among the maxillary second molars, 24 (80%) and four (13%) teeth had two and three MB canals, respectively. The MB2 canal was detected at the chamber floor in 70% and 61% of the maxillary first and second molars, respectively. At 1 mm depth, the MB2 canal was found in 15% and 18% of the maxillary first and second molars, respectively. At 2 mm depth, the MB2 canal was found in 3% and 18% of the maxillary first and second molars, respectively. The remaining teeth had the MB2 canal at levels deeper than 2 mm. The MB2 canal was detected in 97% and 93%% of maxillary first and second molars, respectively. Among them, the MB2 canal could be immediately detected in 70% and 61% of the maxillary first and second molars, respectively, once the pulp chamber is exposed. However, the rest of the MB2 were observed at deeper levels in the root and this requires troughing preparation in the chamber floor.

## 1. Introduction

Endodontic therapy relies on several factors that collectively contribute to its success. However, root canal preparation remains one of the most crucial determinants because the efficacy of subsequent treatment steps is highly dependent on its quality and completeness [1]. Unfortunately, due to the complexity of root canal anatomy, it can be difficult to visualize on two-dimensional radiographs. Consequently, it is not uncommon for canal orifices or even entire canals to be overlooked in the process of preparation [2, 3], leading to either incomplete treatment or even failure of the root canal treatment [2, 3]. Therefore, the analysis of internal anatomy is important for the identification of all root canals in addition to thorough chemomechanical debridement [4].

The root canals of the maxillary first and second molars have been described as having the most intricate morphology of all maxillary teeth [5–7]. This is attributed to the high prevalence of a second mesiobuccal canal (MB2) in their mesiobuccal roots that is highly variable in its location [8]. Failure to locate MB2 in maxillary molars has been associated with an increased treatment failure rate [2, 3].

Extensive research has been done to investigate the prevalence of MB2 in maxillary molars both in vitro and in vivo using one or more methods of examination [4, 5, 9–12]. Results varied considerably between different studies with higher incidence of MB2 detected in vitro, where techniques such as clearing technique and sectioning method have been utilized to visualize the root canal system. Recently, however, cone beam computed tomography (CBCT) has been used extensively in such studies and has replaced in large the traditional techniques [13]. Being a three-dimensional rather than a two-dimensional radiographic technique, it is a powerful tool that provides the unique ability to examine the root canal system in slices of any desirable thickness or as CT reconstructions without the need to section or destroy the tooth [13]. The latest addition to the radiographic methods used to study root canal morphology in vitro is the use of microcomputed tomography (micro-CT). Because of its ability to produce images of higher resolution than those produced with CBCT, micro-CT is now considered the gold standard in studying root canal morphology. Multiple studies reported on the morphology of the mesiobuccal root of maxillary molars using micro-CT [6, 14–16].

The worldwide prevalence of MB2 using CBCT was found to be 73.8%, with a range from 48% to 97.6% [17]. In the Saudi population, studies reported the incidence of MB2 to range between 23.3% and 70.6% in the maxillary first molars [12, 18–21] whilst one study found the incidence in maxillary second molars to be 19.8% [12]. Although the incidence of MB2 in maxillary molars obtained from the Saudi population has been reported in the past [12, 18, 20, 21], the use of micro-CT to investigate the root canal system of maxillary first and second molars in this population has not been done previously. Therefore, the aim of this in vitro study was to investigate the root canal configuration and to determine MB2 detectability in maxillary first and second molars obtained from a Saudi population, using micro-CT.

## 2. Materials and Methods

### 2.1. Sample Selection

Ethical approval for this experiment was obtained from the institutional review board of King Abdullah International Medical Research Center (RC10/043). Maxillary first and second molar teeth, with intact mesiobuccal roots and fully formed apices, were randomly selected from a pool of teeth extracted from the Saudi population. Teeth were cleaned and stored immediately in normal saline solution.

### 2.2. Micro-CT Scanning

The maxillary first (*n* = 35) and second (*n* = 30) molars were scanned at an isotropic resolution of 13.6 microns with micro-CT technology by using a SkyScan 1172 (Bruker microCT, Kontich, Belgium). Each tooth was mounted on the computer-controlled turntable of the micro-CT system such that the X-ray beam was perpendicular to the root surface. The X-rays were generated at 89 kV and 112 *μ*A. Scanning was performed with 180-degree rotation around the vertical axis with a camera exposure time of 1200 milliseconds and a rotation step of 0.54 using an Al 0.5 mm filter.

The acquired images were reconstructed into cross-sectional slices with a beam hardening correction of 10% and ring artifact correction of 5 by using NRecon software (Bruker microCT). CTAn software (Bruker microCT) was used to present the cross-sectional slices in the mesiobuccal root.

The slices were observed starting from the pulp chamber floor level until the apex. The number of canals and number of portals of exit were recorded. In case of having more than one canal, the level of extracanal detection, canal configuration, and presence of isthmus were analyzed. The presence of extracanal was categorized based on the level they were first detected at as follows: 0 level (chamber floor), 1 mm level (1 mm apical from the floor), 2 mm level (2 mm apical from the floor), and >2 mm level (more than 2 mm apical from the floor). The mesiobuccal root canal configurations were classified according to Pomeranz et al. as independent, confluent, and fin [22]. The independent canal system is defined as distinct root canals extending from the pulp chamber to the apex. The confluent canal system is defined as canals that join and end as a single foramen. The fin canal system is defined as joined canals that end as separate foramina.

The images were analyzed by three calibrated endodontists trained on the use of the software. In the event of discrepancy, a consensus was reached by discussion between the three endodontists.

## 3. Results

**Figure 1:**
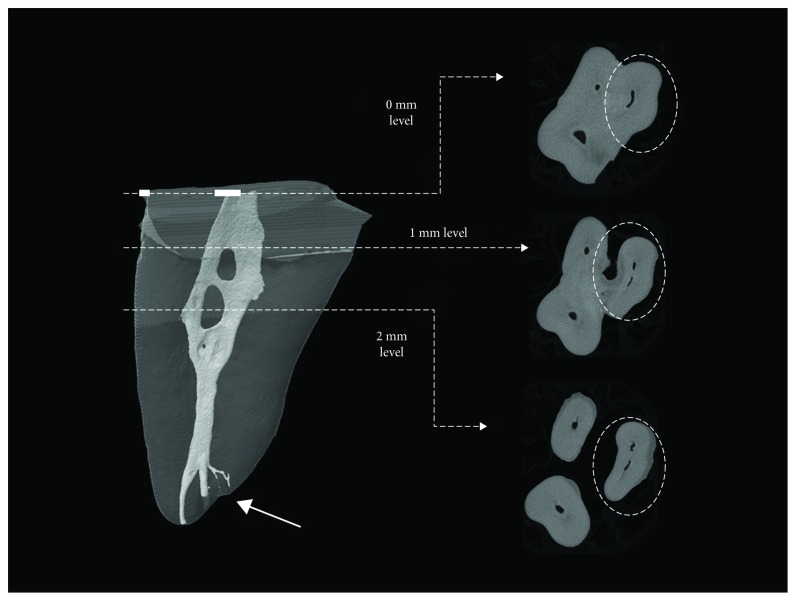
Representative three-dimensional image of mesiobuccal root reconstructed with NRecon software. The root has “confluent” canal configuration with isthmuses in the coronal and middle thirds. At the “0 mm” level, the mesiobuccal root has 1 canal space that becomes 2 canals as shown at “1 mm” and “2 mm” levels (dashed circles). The second mesiobuccal canal can be revealed with 1 mm troughing. The apical delta has 4 portals of exit (arrow).

The reconstructed mesiobuccal roots and their cross sections were analyzed (Figure 1). Among the maxillary first molars, 28 teeth (80%) had two MB canals and six teeth (17%) had three MB canals. Among the maxillary second molars, 24 (80%) and four (13%) teeth had two and three MB canals, respectively (Table 1). More than one portal of exit was found in 77% and 83% of the maxillary first and second molars, respectively (Table 1).

Further analysis was performed on the mesiobuccal roots which have more than one canal. The MB2 canal was detected at the chamber floor in 70% and 61% of the maxillary first and second molars, respectively. At 1 mm depth, the MB2 canal was found in 15% and 18% of the maxillary first and second molars, respectively. At 2 mm depth, the MB2 canal was found in 3% and 18% of the maxillary first and second molars, respectively. The remaining four maxillary first molars and one maxillary second molar had the second MB canal at levels deeper than 2 mm (Table 2).

Canal configuration was found to be mainly “confluent” followed by “independent” and then “fin” type. Canal isthmuses were found anywhere along the mesiobuccal roots in more than 97% of the tested molars, mainly in the coronal third (Table 2).

## 4. Discussion

The first critical step in canal debridement is canal identification. The high prevalence of MB2 in maxillary molars, which are often undetected and missed during root canal treatment, creates a significant clinical challenge [2, 3]. The presence of missed canals in endodontically treated teeth using CBCT was found to increase the prevalence of an apical radiolucent lesion by more than 4 times [23]. This difficulty in canal identification can be related directly to the variations and complex morphology in the mesiobuccal root of maxillary molars, which have been demonstrated dating back to 1921 [24], where a root with a tapered canal and a single portal of exit was stated as the exception rather than the rule. The detection of additional canals in the mesiobuccal root of maxillary molars has been investigated both in vitro [5, 9] and in vivo [10–12, 25], and the MB2 was found to range from 18.6% [26] to 96.1% [27]. The discrepancy in the reported MB2 prevalence in different studies can be attributed to the variable methods of examination used for their detection and the different races and ages evaluated. In clinical studies in which multiple examination methods were used, the likelihood of detecting the MB2 was increased [4, 25]. In the current study, the small number of the maxillary first and second molars tested is a limitation. Difficulty in finding extracted intact molars nowadays is encountered. Nevertheless, the reported prevalence of MB2 of maxillary first molars tested in our study was similar to those found in other countries such as England, Syria, South Africa, and Belgium [17].

Nowadays, the 3D imaging technology can detect the MB2 canal more frequently with a direct relationship found between the reliability in detecting MB2 and the increase in imaging resolution [28]. Micro-CT is now considered the gold standard in lab-based tooth morphology studies [6, 9, 15, 16]. Its advent has provided the clinician with increased insight into canal morphology and facilitated interactive image manipulation and enhancement to reconstruct and visualize the area of interest as a 3D volume from various angles. Despite that, using CBCT technology for canal detection is potentially more practical where age, race, and gender can be highlighted besides the fact that a large number of patients undergo this imaging modality during the course of their dental treatment, which can additionally be utilized in canal morphology studies. However, the CBCT does not show as high resolution as micro-CT technology which necessitates results with fine details.

Al-Shehri et al. studied CBCT images taken for Saudi patients and found the MB2 in 64.6% of maxillary first molars with different configurations [21]. Recently, an elegant multicenter CBCT study has evaluated the prevalence of MB2 canals in maxillary first molars across 21 countries and found to be 73.8%, with a range from 48% to 97.6% [17]. In the current study, micro-CT technology showed the incidence of MB2 to be 97% and 93% in the maxillary first and second molars, respectively. Of note is that the reported prevalence of MB2 in the current study was similar to those found in other countries such as England (91.2%), Syria (95.2%), South Africa (95.6%), and Belgium (97.6%).

The MB3 canal was observed in 17% and 13% of the maxillary first and second molars, respectively. Past studies showed that MB3 was present in the mesiobuccal root of maxillary first and second molars in small percentages, in the ranges of 0.5-12.5% [5, 29] and 0.6-4% [7, 30], respectively.

One potential reason that explains why MB2 canals are frequently missed is that these canals often occur at levels deeper than that of the chamber floor [8]. The present results might provide dental practitioners with the fact that extra MB canals are found at deeper levels in the maxillary molars. The MB2 canals, when present, were detected at the chamber floor level in 70% and 61% of the tested maxillary first and second molars, respectively. However, the rest were detected at deeper levels which addresses the importance of performing more dentinal removal at the chamber floor. This technique was discussed in several articles which proved that dentinal troughing significantly improved effectiveness of detecting MB2 in maxillary molars [8, 25, 31]. Based on our results, selective dentinal removal (troughing) of up to 2 mm will allow the clinician to locate the MB2 canals in 88% and 97% of the tested teeth in total (Table 2).

Failure to treat extracanals is a risk factor for persistent apical periodontitis since it might harbor microbial biofilm [23]. Therefore, it is prudent to better understand the tooth root morphology by means of 3D imaging technology to anticipate the challenge of root canal treatment. It has been reported that when extracanals in the mesiobuccal root are suspected, the access cavity should be modified to locate their potential canal orifices [32, 33]. Based on the present results, the cavity modification should be limited not only to horizontal extension of the cavity but also to apical extension which is represented as floor troughing, to expose the buried canal orifices in the mesiobuccal root area, if present [32, 33]. However, the chamber floor should not be troughed more than 2 mm to avoid jeopardizing the tooth integrity and possible tooth perforation [8].

Apical delta is known to be an intricate system of cavities in which numerous passages of blood vessels and nerves branch from the pulp cavity to the root apex [34]. Clinically, this region might get inflamed or infected and contribute to the initiation of periradicular disease [35]. This disease, along with difficulty in cleaning the apical deltas, might compromise treatment outcome [35]. In maxillary molars, the presence of apical delta in the mesiobuccal root is much higher than those in the distobuccal and palatal roots [16]. The current results found the apical deltas in 77% and 83% of MB2 in the maxillary first and second molars, respectively. These percentages are much higher than the previously stated findings where they could not detect more than 25% [5, 16]. This difference might reflect variations in methodology and population characteristics.

The majority of MB root canal configurations were found to be “confluent” (>60% of the tested roots), followed by “independent” and then “fin” type. Consistently, a previous study showed that more than half of the MB roots exhibited a confluent canal configuration [17]. These configurations along with the presence of apical deltas and canal isthmuses require the use of magnification tools and disinfection solutions and techniques to overcome the pitfalls that might be encountered during endodontic treatment.

## 5. Conclusion

The MB2 canal, when present, could be immediately detected in 70% and 61% of the maxillary first and second molars, respectively, once the pulp chamber is exposed. However, the rest of the MB2 were observed at deeper levels in the root and this requires troughing preparation in the chamber floor.

## Figures and Tables

**Table 1:** Number of canals (percentage) and portals of exits in the mesiobuccal root of maxillary first and second molars.

	Number of canals			Portals of exit		
	1	2	3	1	2	>2
First molar (*n* = 35)	1 (2.9)	28 (80)	6 (17.1)	8 (22.9)	11 (31.4)	16 (45.7)
Second molar (*n* = 30)	2 (6.7)	24 (80)	4 (13.3)	5 (16.7)	11 (36.7)	14 (46.6)

**Table 2:** Distribution (percentage) of the extracanal observation level, canal configuration, and presence of isthmus in the mesiobuccal root of maxillary first and second molars.

	Extracanal detection				Canal Configuration			Presence of isthmus			
	0 mm	1 mm	2 mm	>2 mm	Independent	Confluent	Fin	Overall	Coronal	Middle	Apical
First molar (*n* = 34)	24 (70)	5 (15)	1 (3)	4 (12)	10 (29.4)	21 (61.8)	3 (8.8)	33/34 (97)	32/34 (94)	27/34 (79)	14/34 (41)
Second molar (*n* = 28)	17 (61)	5 (18)	5 (18)	1 (3)	8 (28.6)	18 (64.2)	2 (7.2)	28/28 (100)	22/28 (79)	14/28 (50)	6/28 (21)

## Data Availability

The experiment's data used to support the findings of this study are available from the corresponding author upon request.
